# Improving the Follow-up Rate for Pediatric Patients (0-16 years) of an Eye Hospital in Nepal: Protocol for a Public Health Intervention Study

**DOI:** 10.2196/31578

**Published:** 2021-10-08

**Authors:** Manisha Shrestha, Gopal Bhandari, Suresh Kumar Rathi, Anirudh Gaurang Gudlavalleti, Binod Pandey, Ramesh Ghimire, Daman Ale, Sajani Kayastha, Daya Shankar Chaudhary, Raghunandan Byanju, Operational Research Capacity Building Study Group

**Affiliations:** 1 Bharatpur Eye Hospital Chitwan Nepal; 2 Indian Institute of Public Health Hyderabad India; 3 See Authors' Contributions

**Keywords:** counseling, follow-up, intervention study, pediatric patients, ophthalmology, public health, Nepal

## Abstract

**Background:**

The follow-up of pediatric patients ensures regular ocular morbidity monitoring and better treatment outcome. Hiralal Santudevi Pradhan Institute of Ophthalmic Science (Bharatpur Eye Hospital [BEH]) noticed that the follow-up rate was only 22% among its pediatric patients. Several factors like lack of awareness and forgetfulness among patients may contribute to a lower number of follow-up visits. Therefore, BEH decided to find if counseling and reminders through SMS text messaging and phone calls would improve the follow-up rates.

**Objective:**

This study aims to evaluate the impact of interventions like counseling and reminder SMS text messaging and phone calls in improving the follow-up rate of pediatric patients.

**Methods:**

This is a public health intervention study being conducted using quantitative analysis. All children (0-16 years) with ocular conditions requiring at least 3 follow-up visits in the study period will be included. In all, 264 participants will be allocated to 3 groups: routine standard care, counseling, and reminders with SMS text messaging and phone calls. In counseling, patients will take part in 20-minute counseling sessions with trained counselors at each visit, and information leaflets will be provided to them. In the reminder SMS text messaging and phone call group, patients will receive an SMS text message 3 days prior and a phone call 1 day prior to their scheduled visits. Patients attending within 2 days of the scheduled date will be considered compliant to follow-up. The proportion of patients completing all the follow-up visits in each group will be assessed. Informed consent will be taken from parents and children. Univariate and multivariate analyses will be conducted.

**Results:**

The ethical approval for this study has been obtained from the Ethical Review Board (ERB) of Nepal Health Research Council (ERB protocol registration #761/2020 P). The data collection was initiated on January, 24, 2021, but due to the COVID-19 pandemic, as of September 2021, we have only been able to enroll 154 of the planned 264 participants (58.3% of the sample size).

**Conclusions:**

This study will reliably document not only the factors associated with follow-up rate through an intervention package (counseling and reminders through SMS text messaging and phone calls) but also the cost effectiveness of the intervention package, which can be applied in all the departments of the hospital.

**Trial Registration:**

ClinicalTrials.gov NCT04837534; https://clinicaltrials.gov/ct2/show/NCT04837534

**International Registered Report Identifier (IRRID):**

DERR1-10.2196/31578

## Introduction

Childhood visual impairment and blindness remains an important public health issue. It is estimated that around 14 million children in the world are blind [1]. The Nepal Pediatric Ocular Disease study found that the prevalence of blindness and visual impairment in the community was 0.068% (95% CI 0.02-0.12) and 0.097% (95% CI 0.04-0.15), respectively [2]. Increasing the global knowledge base for planning for childhood eye care services is a top priority in order for children with visual impairment to realize their full visual potential [3]. Follow-up of pediatric patients is important for their regular ocular morbidity monitoring, especially for amblyopia management [4]. Bharatpur Eye Hospital (BEH) observed that there was poor adherence to follow-up visits. An exploratory observation of data of the first week (January 1, 2010, to January 7, 2019) revealed that the follow-up compliance was very low among children aged 0 to 16 years in the pediatric department. Among the children advised for follow-up, only 22% were found to have come for at least 1 follow-up visit. A problem analysis showed that a lack of awareness in children and their parents regarding the importance of follow-up and patients forgetting dates of the follow-up visit (usually when there is long gap for follow-up) may be the major contributing factors for poor adherence to follow-up.

A study from India revealed that distance and cost were major barriers, as was the inability of the eye care center to communicate the importance of follow-up [5]. Another study done in Nepal found poor follow-up rates for patients following cataract surgery, which, however, improved after implementation of a high-quality pediatric counseling service, follow-up program, tracking system, and cell phone reminders [6]. Many studies have compared different methods of reminder options like telephone calls, email, and SMS text messaging in order to improve the compliance to follow-up [7-10]. Hence, a public health intervention study has been planned to estimate the effect of intervention package (counseling and reminder SMS texts and phone calls) for improving the follow-up rates in pediatric patients.

The primary objectives are to determine if parental counseling has an impact on adherence to follow-up and to determine if reminders through phone calls and SMS texts increase the follow-up rate in pediatric patients. The secondary objective is to assess the cost-effectiveness of counseling and reminder SMS texts and phone calls. We hypothesize that counseling will increase the follow-up rate from 22% to 50% and reminders through phone calls and SMS texts will increase the follow-up rate from 22% to 25% at the site of the study (mentioned in the next section).

## Methods

### Study Design and Setting

This public health intervention study will be conducted at Hiralal Santudevi Pradhan Institute of Ophthalmic Sciences which includes 13 satellite clinics and 1 base hospital (Bharatpur Eye Hospital [BEH]). The study participants will be enrolled only from the pediatric department of BEH, a centrally located tertiary eye hospital in the Chitwan district of Nepal. All pediatric patients 0 to 16 years of age meeting the inclusion criteria will form the study population. In the pediatric department of BEH, children aged 0 to 16 years are being examined as per the hospital policy.

The inclusion criteria are all pediatric patients 0 to 16 years of age advised for at least 3 follow-up visits within a 6-month period at BEH, parents or guardians of children who own a mobile phone or have daily access to a phone and are able to use the SMS text feature on these phones, and parents willing to enroll their children in the study.

The exclusion criteria are children who require fewer than 3 follow-up visits, children with ocular conditions requiring daily follow-up visits (because it is not feasible to send the reminder phone call and SMS text for daily follow-up), children who have already been treated and are under regular follow-up, children with ocular conditions requiring follow-up beyond 6 months, parents or guardians of the children who are not willing to participate in the study, and those who have been called but do not answer after 3 attempts

### Sample Size

Bonferroni correction (level of significance *P*=.05; k=3 comparisons) was adopted for sample size calculation with consideration to the proportion of attendance in counseling (p1) and reminder SMS texts and phone calls (p2) as 50% (0.5) and 25% (0.25), respectively. An estimated sample size of 264 pediatric patients will be targeted with an adjusted significance level of .02, 80% power, and 10% loss to follow-up.

### Intervention Package

#### Counseling Group

In this group, after the children have been examined, a treatment plan and follow-up schedule will be advised by the pediatric ophthalmologist to the parents or guardians along with the child. The parent or guardian and the child will receive counseling from a trained counselor (SK) as per the set counseling protocol in every follow-up visit where the disease-specific leaflet will be used as a counseling tool, a copy of which will also be handed over to them. A counseling protocol for the common ocular conditions has been designed by the research team. The counselor will be oriented and trained as per the counseling protocol a week before the participants are enrolled in the study. Children, along with their parent or guardian, will be receiving counseling irrespective of patient age, parental education, ocular conditions, and other factors. If more than one guardian or both mother and father are accompanying the child, both will be included in the counseling session. The counselor will deliver verbal counseling for all participants in all follow-up visits irrespective of the ocular conditions and other factors. The counseling session for all participants will last for 20 minutes.

#### SMS Text and Phone Call Reminder Group

In this group, after the children have been examined and a treatment plan and the follow-up have schedule been advised, they will be discharged from the department. They will receive an SMS text 3 days prior to and a phone call 1 day prior to their scheduled follow-up visits. Text messages will be sent until it is confirmed that the sent message has been received. In the case of message delivery failure, it will be sent again 3 more times to ensure successful delivery. If the SMS text is not delivered after 3 attempts, then a message will be sent to the next phone number (if available) as recorded in the proforma during the interview. A phone call will be deemed to be completed once it is received by the respondent; calls will be repeated at least 3 times if the phone is not answered in the first instance. If the call is not answered even after 3 attempts, the participant will be excluded from the study.

The assistant (DA) will be responsible for making the reminder SMS and calls, making the records, and tracking the follow-up patients. The standard text and the call format have been developed in the local language (Nepali).

#### Routine Standard Care Group/Control Group

In this group, the children will undergo visual acuity testing and refraction by an optometrist. The pediatric ophthalmologist will perform detailed ocular examination and advise necessary investigations to diagnose and formulate a treatment plan. Basic counseling will be done by the consultant regarding the ocular condition, treatment, and need for follow-up. No additional counseling or reminders will be performed for these patients. They will be discharged from the department and advised for follow-up as per the hospital protocol. The follow-up schedule as per the hospital protocol for common ocular conditions is shown in Table 1.

**Table 1:** Schedule for follow-up for different ocular conditions.

Ocular condition	Recommended guidelines for follow-up [11]
Congenital naso-lacrimal duct obstruction	Once every 4 weeks
Keratitis	Depending on severity, at least once a week
Allergic conjunctivitis	Once every 2 weeks
Ocular injury	Depends on severity of injury, daily to once a week
Pediatric cataract	Once every 2 weeks
Strabismus	Once every 2 weeks
Amblyopia	Once every 4 weeks
Retinopathy of prematurity	Once every 2 weeks

### Outcomes

The primary outcome is the proportion of children completing all 3 follow-up visits in the routine standard care group, counseling group, and reminders through SMS texts and phone call group.

The secondary outcomes include the effect of parental or guardian education status in compliance to follow-up, the visual status of the children in subsequent follow-up visits, the effect of traveling distance and cost in compliance to follow-up, and the cost-effectiveness of the intervention.

### Independent Variables

The independent variables will include the following sociodemographic factors: gender, age, ethnicity, educational status, occupation of parent or guardian, attendant relationship with the child (of parent or guardian attending with the child), distance traveled, and cost for the travel to the hospital.

### Study Procedure

All participants will be enrolled in the study after fulfilling the inclusion criteria and administering informed consent. Recruitment will be conducted over a 6-month period. Participants will be assigned a unique code which will be a 6-digit number generated by using the last 3 digits of the patient’s medical record number and the last 3 digits of the parent’s or guardian’s mobile phone number. The participants will be entered into 3 groups, such that the first patient will be enrolled to the routine standard care group, the second to the counseling group, and the third to the group of SMS texts and phone call reminders. The same sequence will be followed for every participant until the required sample size has been attained. Before the actual start of the recruitment, 2 dry runs were conducted to understand the flow of the patients and logistics of the study.

After registration, the patient will be guided by the Outpatient Department (OPD) facilitator to the vision and refraction room and then to the consultant room. After ocular examination, treatment and follow-up advice, the consultant will paste a colored sticker on the patient’s file to indicate the potential study participant. There will be a separate and dedicated place for the pediatric ophthalmologist, assistant, and the counselor (Multimedia Appendix 1).

Patients with a colored sticker on their OPD file will be referred to the assistant room. The assistant will take the written consent from the children in an assent form (if 9 years or older) and from their parents or guardians in a consent form after explaining the study and group and selection to them (Multimedia Appendix 2 and 3).

A unique number will be allotted to the patient (generated through the medical record number and mobile phone number of the patient). The unique ID of the participants will be recorded in the proforma and on the examination file by the assistant. He will fill all the details in the proforma (Multimedia Appendix 4) and also enter the details in a Microsoft Excel sheet.

The patients assigned to counseling will be sent to the counselor. The counselor will conduct a counseling session based on the ocular condition and follow-up plan. Counseling will be done as per the prescribed counseling protocol. After counseling, the counselor will provide the information leaflets, and the patient will be asked to visit the optical dispenser or pharmacist.

For patients assigned to the SMS text and phone call reminders or control groups, the necessary details will be filled in the proforma and Excel sheet before they are discharged by the assistant.

### Identifying the Follow-up Patients

The study participants will have a colored sticker attached to their OPD card during their first visit. The assistant will provide a unique code to the study participants, which will be written on top of the colored sticker attached on their OPD card. Thus, during the follow-up visit, the participants will be easily identified by this colored sticker with the unique code in their OPD card. The assistant will match the unique code of the patient with the computer records and find out the study group of the patients in each follow-up visit.

In case the patient card is lost or if the patients comes with a new OPD card during follow-up, the earlier registered medical record number will not match with the new medical record number. In this case, the patient’s identity will be confirmed by matching the patient’s name, gender, age, guardian’s name, and guardian’s phone number.

### Compliance to Follow-up

The participants’ first visit to the hospital and the scheduled 3 follow-up visits need to be completed for them to be considered compliant to the follow-up. Only those participants who complete the first follow-up will be considered for second follow-up, and only those coming for second follow-up will be considered for third follow-up [6]. The patient will be considered compliant to follow-up if he or she comes within the window period of 2 days. The rescheduling of the next follow-up date will be calculated from the attended date as per the follow-up schedule for each ocular condition.

The purpose of observing compliance to follow-up is to determine the impact of counseling and reminders through SMS texts and phones call for the increment of the proportion of children completing their 3 follow-up visits based on the developed proforma and to find out the significant proportion in the follow-up rate between the 3 different groups.

### Record Maintenance

Records of the participants from the first visit to all the follow-up visits will be maintained in the department by the assistant. The details will be entered for each participant as per the proforma. If both parents accompany the child, only the mother’s detail will be included. However, when both parents accompany the child but only the father has access to a mobile phone, his details will be recorded.

The record of the patients will be kept even if they come for follow-up beyond the set window period (2 days); however, in this case, they will not be considered compliant to follow-up.

The assistant will also assign the patients to a group (regardless of whether they receive an intervention) as per the study procedure. A follow-up recording file (3 in number, 1 each for routine standard care, counseling, and reminders through SMS texts and phone calls) will be maintained. These follow-up details will also be entered in the Excel sheet on the same day as a backup in case the paper records are lost or destroyed. The entry of all the details from the proforma will be entered and stored in an Excel sheet by the assistant. The status of record maintenance will be regularly monitored by the ophthalmic officer (BP). The assistant will also be responsible for tracking the follow-up visits and updating the schedule for executing the reminder SMS texts and phone calls (Multimedia Appendix 5).

### Data Collection and Management

In order to obtain the required data from the participants, a proforma has been designed based on variables pertaining to sociodemographics and follow-up. The proforma has been developed in the English language, and the sequence of questions will be carefully looked into. Questions are arranged in the following manner: the first pertain to identification, are followed by those pertaining to sociodemographic information, and are concluded by questions pertaining to follow-up points.

#### Training of the Team

All team members will receive training on the study process. The principal investigator (MS) or coprincipal investigator (GB) will brief the team on the process during the training session, including on the consent of the participant, being polite and considerate, the standardized delivery of questions, and the noting of the responses. During the training sessions, each and every question will be explained to the team, and queries will be addressed.

#### Data Editing

Data will be submitted as early as possible to the investigator team so that they can edit the proformas and make them ready for data entry.

#### Data Entry and Validation

The data entry program will be designed in Excel. The study optometrist and coprincipal investigator will do the data entries. Corrections will be made where required by the study’s ophthalmic officer, who will compare the computer data file with the original proforma.

#### Data Monitoring and Confidentiality

The data monitoring will be performed by an internal monitoring committee on a weekly basis. All the information collected will be stored only with the investigators. The publications will not reveal any private information of any of the participants.

#### Data Analysis

Data will be processed and analyzed using Excel (Microsoft) or Epi Info (Centers for Disease Control and Prevention).

#### Univariate Analysis

Univariate logistic regression analysis will be conducted by comparing 2 variables for each variable of interest using odds ratios (ORs) and their 95% CIs. The likelihood ratio test will be used to estimate ORs and 95% CIs for all associations of interest.

#### Multivariate Analysis

Multivariate logistic regression analysis will be performed to adjust for the simultaneous effects of multiple factors on the outcome variable. The criteria for inclusion of factors in the multivariate analysis are to include all variables from the univariate analysis with a *P* value of .1 along with all the variables of known importance. To assess the importance of each variable included in the model, the Wald statistic for each variable will be used.

Before the multivariate analysis is conducted, the association among independent variables will be checked by a chi-square test. For independent variables having more than 2 categories, dummy variables will be created. All the variables meeting the above selection criteria will be entered one by one, starting with the highly significant factors from the univariate analysis. The overall significance of independent variables in the model will be assessed by likelihood ratio test (G statistic). Selection of the final model will be based on parsimony, biological interpretability, and statistical significance. The parameters of the logistic regression model will be estimated by the maximum likelihood method. The adjusted ORs and their 95% CIs will be computed using the estimates of parameters of the final model. The dependent variable will be dichotomous: either compliance with the follow-up instruction has increased, or it has remained changed (no difference) from before the intervention study to after it. The final model will be tested for goodness of fit by Hosmer- Lemeshow chi-square (*Χ*^2^) statistic. *P* values will be noted to assess the model fit. Imputation and intention to treat analysis will also be conducted (if required).

#### Cost-Effectiveness Analysis of Counseling and Reminder SMS Texts and Phone Calls

The total costs involved in implementing the interventions will be compared with the attendance of follow-up patients and the revenue generated through their follow-up sessions. The intervention costs include the salary for the team members. In addition to the costs for the phone calls and reminder SMS telecommunication charges, the cost for equipment, stationery, and the charges for utility services like water and electricity will also be included as the cost for implementing interventions. Cost effectiveness will be analyzed separately for counseling and reminders (phone calls and SMS texts) by calculating the total cost incurred per attendance of follow-up in each intervention group and the total revenue generated through the fee for the OPD ticket, investigations, medicines, and glasses provided during the follow-up visits.

### Ethical Approval

The ethical approval has been obtained from Ethical Review Board (ERB) of the Nepal Health Research Council (ERB protocol registration #761/2020 P). The data collection was initiated on January 24, 2021, but due to the COVID-19 pandemic, as of September 2021, we have only been able to enroll 154 of the 264 participants (58.3% of the sample size).

## Results

Descriptive statistics will be presented, and demographic variables will be summarized by computing means with SD for continuous variables and proportions for categorical variables. Associations between sociodemographic variables and compliance rates will be presented. The associations will be computed using categorical variables. Two dry runs were conducted to assess the logistics and flow of participants. The first dry run was conducted by taking only 10 patients who were not the actual study participants, but the second dry run was conducted with the actual participants as per the inclusion and exclusion criteria. A total of 38 study participants were enrolled for a duration of 1 month for the second dry run. Of these participants 13, 13, and 12 were divided into the standard care group, counseling group, and reminder with SMS texts and phone call groups, respectively. The mean age of the children was 3.71 (SD 0.61) years, with a range of 0.07 to 14 years. There were more male children 24 (63.16%) as compared to female children 14 (36.84%). Most of the children were of Aryan descent (63%), and more than 80% of the children were accompanied by their mother. Only 7 out of the 38 patients attended the follow-up visit. All those who attended have attended only the first follow-up visit. Three participants were from reminder group, two were from the counseling group, and two were from the standard care group. Out of the 38 children, 14 (36.8%) were diagnosed with congenital naso-lacrimal duct obstruction and 7 with amblyopia, with the others having conjunctivitis, eye injury, corneal ulcer, or squint.

## Discussion

This study will reliably document not only the factors associated with follow-up rate through an intervention package (counseling and reminders through SMS texts and phone calls) but also the cost effectiveness of the intervention package. Compliance to follow-up visits is important for better outcomes of treatment for any condition. It is equally important for children with visual problems to comply with the follow-up so that they can receive complete treatment and be prevented from going permanently blind in some cases. Different studies have shown varied results regarding the effect of these interventions. Follow-up rates have found to be improved with these types of intervention in some studies while other studies show no significant improvement with the interventions [12-17]. One intervention may work better than others in some studies. We are testing these interventions to improve the follow-up rate of pediatric patients with ocular problems. Loss or noncompliance with the scheduled follow-up visits by patients who have been advised to come for follow-up is a major issue in many areas of clinical practice. Noncompliance has been a major problem with pediatric patients undergoing treatment at the Pediatric Ophthalmology Department at BEH, where only 22% of those advised attended the follow-up. Therefore, this study is attempting to determine which intervention strategy would work better for this problem. Any intervention found effective will be applied to other departments and will be implemented as the hospital policy in the future.

## Appendix

Multimedia Appendix 1 Patient flowchart.

Multimedia Appendix 2 Assent to participate in the study.

Multimedia Appendix 3 Consent to participate in the study.

Multimedia Appendix 4 Proforma for the ocular condition.

Multimedia Appendix 5 Format of SMS texts and phone calls.

## Abbreviations

BEH: Bharatpur Eye Hospital
ERB: Ethical Review Board
OPD: Outpatient Department
