# Supplementary material for: Improving the Follow-up Rate for Pediatric Patients (0-16 years) of an Eye Hospital in Nepal: Protocol for a Public Health Intervention Study
Source: JMIR Res Protoc. 2021 Oct 8;10(10):e31578. doi: 10.2196/31578 (PMC8538025; doi:10.2196/31578)
Supplement: Multimedia Appendix 1 [file resprot_v10i10e31578_app1.docx]

**Patient flow chart**

Patient enter to Hospital

0-16 years age group patient go to pediatric department

Registration section

Get registered with name, age, sex, address and contact entered in IHMS and receive hospital id card

-

Patient are directed to the Vision and refraction room (Number - 15) by OPD facilitator

Consultant room (Room No.16)

Patient examined, treatment and follow up advised and color sticker attached for eligible participants. OPD facilitator take consent and send to assistant room. Non study participants discharged as usual.

--

-

-

Assistant room (14)

Check the pt. group using the unique code on the colored sticker. Send participants of counselling group to the counselor and discharge other participants (standard care and reminder SMS and phone calls)

For pt. with new file, check with the records in excel and conform if he/she is the study participants

Update the records of the follow up patients

Maintain the records of reminder SMS and call schedule and make reminders accordingly.

Registration room

Patient gets old file on the basis of hospital ID card

If patient took the examination file with him/her or lost, then he/she will be registered as new patient and gets new MR no.

Assistant room (14)

Provide unique code to the participant (written on the coloured sticker), fill the proforma and enter in excel sheet, maintain record of the follow up schedule and distribute the participants to study groups.

Direct counselling participants to counselor (18) and discharge other participant(standard care and reminder SMS and phone calls) for optical or pharmacy as advised. Make reminders SMS and phone call as per the schedule.

Counselor room (18)

Counseling for parents/guardian with child

Similar counseling as first visit and content as per the protocol

**Follow up visit**

Consultant room (16)

Patient that come with the old file and with sticker attached are send to assistant room.

If patient come with new file but if consultant suspect he/she is study participants sent to assistant room with sticker attached

Counselor room (18)

Counseling for parents/guardian with child (20 minutes session) as per the standard counseling protocol designed and provide information leaflets
