# Supplementary material for: Improving the Follow-up Rate for Pediatric Patients (0-16 years) of an Eye Hospital in Nepal: Protocol for a Public Health Intervention Study
Source: JMIR Res Protoc. 2021 Oct 8;10(10):e31578. doi: 10.2196/31578 (PMC8538025; doi:10.2196/31578)
Supplement: Multimedia Appendix 3 [file resprot_v10i10e31578_app3.docx]

**Consent to participate in the study ‘Improving the follow up rate for Pediatric Department at Bharatpur Eye hospital, Bharatpur, Chitwan, Nepal’**

Nepal Netra Jyoti Sangh, Bharatpur Eye Hospital, Bharatpur, Chitwan, Nepal

No. Date:

Thank you for considering to participate in the research conducted by Bharatpur Eye Hospital with guidance from Indian Institute of Public Health (IIPH) Hyderabad and SEVA foundation.

In this study Dr Manisha Shrestha and her team are trying to find out the effectiveness of counseling and reminders through phone calls and SMS to improve the follow up rate among children attending pediatric department of Bharatpur Eye Hospital. We are planning to include a total of 264 children who will be distributed into three groups.

If you choose to participate in the study you and your child will be asked some of your details included in the research proforma after the ocular examination and you will be assigned to either of any 3 group. There is no any risk and you will not feel any discomfort during the study procedures.

All efforts will be made to keep all of your information in the strictest confidentiality by restricting access to your information to key study staff and identifying information from your data will not be disclosed.

There will be no cost incurred upon you for your participation, and you will not receive any money for your participation in this study. Participation is voluntary and you are free to decide to stop participating in this study at any time.

If you have additional questions or concerns about this study or your participation in it, please see study Principal investigator in person at the Bharatpur Eye Hospital or call 0097-56-520333.

Date: …………………..

………………………………………..

Name and signature of Parent/guardian
