# Supplementary material for: Improving the Follow-up Rate for Pediatric Patients (0-16 years) of an Eye Hospital in Nepal: Protocol for a Public Health Intervention Study
Source: JMIR Res Protoc. 2021 Oct 8;10(10):e31578. doi: 10.2196/31578 (PMC8538025; doi:10.2196/31578)
Supplement: Multimedia Appendix 4 [file resprot_v10i10e31578_app4.docx]

**Proforma for the ocular condition**

**Part one**

1. **Unique ID ……………………….**
2. **Serial number …………………….**
3. **MR no……………………….**
4. **Date………………………**
5. **Study group-**

1. Routine Standard Care Group

2. Counseling Group

3. Reminder SMS and Phone call Group

1. **Child and guardian demographics**

i. Name of child:-

ii. Age of child:-

iii. Sex of child:- 1. Male/ 2. female

iv. Parents (if both parents then mother)/guardian name:-

v. Parent accompanying child- 1. Mother/ 2. Father/ 3. both

vi. Guardian relation if not parents– 1. uncle/2. aunt/3. sister/4. brother/5. grandparent/6.family friend

vii. Address-

viii. Ethnicity-1. Aryan/2. Mongolian/3. others (specify)

ix. Educational status: Specify.

x. Occupation: 1. Household/ 2. Farmer/ 3. Teacher/ 4. Medical field/ 5. Service /6. labour/ 7. unemployed/ 8. Retired 9.Others (specify)…………………..

xi. Do parent/guardian have phone with valid number- 1.Yes /2.No

xii. Phone number of parent/guardian:-

1. Land line………………………………………2. Mobile no1………………………………3. Mobile no2…………….................

xiii. Distance of hospital from home (in kms)/time taken to reach the hospital…………

xiv. Cost of two way travel per person:

**Part two**

**Details of the child regarding follow up and ocular examination**

1. **Follow up date**

| First follow up | | Second follow up | | Third follow up | |
| --- | --- | --- | --- | --- | --- |
| Advised date | Attended date | Advised date | Attended date | Advised date | Attended date |
|  |  |  |  |  |  |

1. **Schedule for reminder SMS**

| Follow ups | First follow up | Second follow up | Third follow up |
| --- | --- | --- | --- |
| Date |  |  |  |

1. **Schedule for phone calls**

| Follow ups | First follow up | Second follow up | Third follow up |
| --- | --- | --- | --- |
| Date |  |  |  |

**10. Follow up visits**

1. First follow up
2. Second follow up
3. Third follow up

**11. History and ocular examination-**

i. Presenting complaint:

ii. Duration of symptoms:

iii. Previous treatment received- YES/ NO, if yes duration of treatment received:

iv. Drug and allergy-

| v. Visual acuity | Visit | Right eye | Left eye |
| --- | --- | --- | --- |
|  | a. Presenting |  |  |
|  | b. 1^st^ follow up |  |  |
|  | c. 2^nd^ follow up |  |  |
|  | d. 3^rd^ follow up |  |  |

vi. Ocular findings

| Visit |  | Right eye | Left eye |
| --- | --- | --- | --- |
| A. Presenting | a. lids |  |  |
|  | b. lacrymal apparatus |  |  |
|  | c. conjunctiva |  |  |
|  | d. cornea |  |  |
|  | e. anterior chamber |  |  |
|  | f. lens |  |  |
|  | g. vitreous |  |  |
|  | h. retina |  |  |
|  | i. optic nerve |  |  |
|  | j. IOP |  |  |
|  | k. Extraocular movement |  |  |
|  | l. ocular deviation(prism dioptre) |  |  |
|  |  | Right eye | Left eye |
| B. 1^st^ follow up | a. lids |  |  |
|  | b. lacrymal apparatus |  |  |
|  | c. conjunctiva |  |  |
|  | d. cornea |  |  |
|  | e. anterior chamber |  |  |
|  | f. lens |  |  |
|  | g. vitreous |  |  |
|  | h. retina |  |  |
|  | i. optic nerve |  |  |
|  | j. IOP |  |  |
|  | k.Extraocular movement |  |  |
|  | l.Ocular deviation(prism dioptre) |  |  |
|  |  | Right eye | Left eye |
| C. 2^nd^ follow up | a. lids |  |  |
|  | b. lacrymal apparatus |  |  |
|  | c. conjunctiva |  |  |
|  | d. cornea |  |  |
|  | e. anterior chamber |  |  |
|  | f. lens |  |  |
|  | g. vitreous |  |  |
|  | h. retina |  |  |
|  | i. optic nerve |  |  |
|  | j. IOP |  |  |
|  | k. Extraocular movement |  |  |
|  | l.cular deviation (prism dioptre) |  |  |
|  |  | Right eye | Left eye |
| D. 3^rd^ follow up | a. lids |  |  |
|  | b. lacrymal apparatus |  |  |
|  | c. conjunctiva |  |  |
|  | d. cornea |  |  |
|  | e. anterior chamber |  |  |
|  | f. lens |  |  |
|  | g. vitreous |  |  |
|  | h. retina |  |  |
|  | i. optic nerve |  |  |
|  | j. IOP |  |  |
|  | k. extraocular movement |  |  |
|  | l. ocular deviation (prism dioptre) |  |  |

12.**Diagnosis……………………………………………..**

13. **Perceived seriousness of the condition by the mother**-

Presenting: 1. mild/ 2. moderate/ 3. severe

14.**Treatment outcome since last visit**

A. At 1^st^ follow up-1. getting worse/ 2. getting better/ 3.no change

B. At 2^nd^ follow up- 1. getting worse/ 2. getting better/3. no change

C. At 3^rd^ follow up-1. getting worse/2. getting better/ 3.no change
