# Supplementary material for: Improving the Follow-up Rate for Pediatric Patients (0-16 years) of an Eye Hospital in Nepal: Protocol for a Public Health Intervention Study
Source: JMIR Res Protoc. 2021 Oct 8;10(10):e31578. doi: 10.2196/31578 (PMC8538025; doi:10.2196/31578)
Supplement: Multimedia Appendix 5 [file resprot_v10i10e31578_app5.docx]

**Format for reminder SMS**

This is a reminder SMS for your child’s upcoming follow up at Bharatpur Eye Hospital which is scheduled after 3 days (date of follow up).

**Format for phone calls**

Namaste, I am from Bharatpur Eye Hospital. I have made this phone call to remind you about your child’s (child’s name) follow up at Bharatpur Eye Hospital which is scheduled for tomorrow. Thank you.
